# Supplementary material for: SyNDI: synchronous network data integration framework
Source: BMC Bioinformatics. 2018 Nov 6;19:403. doi: 10.1186/s12859-018-2426-5 (PMC6219086; doi:10.1186/s12859-018-2426-5)
Supplement: Supplementary file 5 — Files of the example presented in Additional file 4. This additional file is a zip package that contains all files used in Additional file 4. Detailed descriptions of each of these files are presented in a README file included in this zip package. (GZ 30411 kb) [file 12859_2018_2426_MOESM5_ESM.gz › SyNDITools/Meme2Fimo/meme_4.9.0_4.tar.gz!/meme_4.9.0/website/html/help_email.html]

MEME Suite - Email & Description Fields


|  |  |
| --- | --- |
|  | MEME Suite The MEME Suite web application requires the use of JavaScript   Javascript doesn't seem to be available on your browser.  - **e-mail address**    Enter the e-mail address where you want the confirmation message to   be sent. Be sure that this is a valid e-mail address.    **Note:**The email address will never be used for any   purpose other than sending you the confirmation message   containing a link to your results.- **Re-enter e-mail address**      This field helps prevent your confirmation message from going     astray.- **description**        This will be included in the confirmation message you will be sent.        This field is optional so you may leave it blank if you wish.        You may only use the following characters in your description: **a-zA-Z0-9:;-\_"()<>%** All other characters will be converted to blanks. |
